# Major depressive disorder with melancholia displays robust alterations in resting state heart rate and its variability: implications for future morbidity and mortality

**DOI:** 10.3389/fpsyg.2014.01387

**Published:** 2014-11-27

**Authors:** Andrew H. Kemp, Daniel S. Quintana, Candice R. Quinn, Patrick Hopkinson, Anthony W. F. Harris

**Affiliations:** ^1^Discipline of Psychiatry, Sydney Medical School, University of SydneySydney, NSW, Australia; ^2^School of Psychology, Faculty of Science, University of SydneySydney, NSW, Australia; ^3^Centro de Pesquisa Clínica e Epidemiológica, Hospital Universitário – Universidade de São PauloSão Paulo, Brazil; ^4^NORMENT, K.G. Jebsen Centre for Psychosis Research, Institute of Clinical Medicine, University of OsloOslo, Norway; ^5^Division of Mental Health and Addiction, Oslo University HospitalOslo, Norway; ^6^Brain Dynamics Centre, Westmead Millennium Institute, University of Sydney – Westmead HospitalSydney, NSW, Australia

**Keywords:** melancholia, non-melancholia, electrocardiogram, ECG, heart rate, heart rate variability, HRV, resting state

## Abstract

**Background:** Major depressive disorder (MDD) is associated with increased heart rate and reductions in its variability (heart rate variability, HRV) – markers of future morbidity and mortality – yet prior studies have reported contradictory effects. We hypothesized that increases in heart rate and reductions in HRV would be more robust in melancholia relative to controls, than in patients with non-melancholia.

**Methods:** A total of 72 patients with a primary diagnosis of MDD (age *M*: 36.26, SE: 1.34; 42 females) and 94 controls (age *M*: 35.69, SE: 1.16; 52 females) were included in this study. Heart rate and measures of its variability (HRV) were calculated from two 2-min electrocardiogram recordings during resting state. Propensity score matching controlled imbalance on potential confounds between patients with melancholia (*n* = 40) and non-melancholia (*n* = 32) including age, gender, disorder severity, and comorbid anxiety disorders.

**Results:** MDD patients with melancholia displayed significantly increased heart rate and lower resting-state HRV (including the square root of the mean squared differences between successive N–N intervals, the absolute power of high frequency and standard deviation of the Poincaré plot perpendicular to the line of identity measures of HRV) relative to controls, findings associated with a moderate effect size (Cohens *d*’s = 0.56–0.58). Patients with melancholia also displayed an increased heart rate relative to those with non-melancholia (Cohen’s *d* = 0.20).

**Conclusion:** MDD patients with melancholia – but not non-melancholia – display robust increases in heart rate and decreases in HRV. These findings may underpin a variety of behavioral impairments in patients with melancholia including somatic symptoms, cognitive impairment, reduced responsiveness to the environment, and over the longer-term, morbidity and mortality.

## INTRODUCTION

Unfortunately, most researchers in psychiatry and psychology express little interest in the mapping of autonomic regulation as a “vulnerability” dimension for various disorders and behavioral problems, although visceral features are often symptoms of the disorders they are treating.

–Porges (2011)

Major depressive disorder (MDD) is associated with reduced resting-state heart rate variability (HRV; Kemp et al., 2010, 2012; Brunoni et al., 2013; see Kemp and Quintana, 2013 for review), and these reductions are inversely associated with disorder severity (Kemp et al., 2010). Heart rate and its variability (HRV) are determined by a variety of physiological factors, although the most prominent of these is the autonomic system. A high level of parasympathetic (vagal) function is both desirable and beneficial as it reflects the capacity for an individual to respond, adapt and regulate responses when required. One can only imagine the consequences of buildings in Tokyo not being sufficiently flexible to withstand an earthquake. The ability to adapt quickly to change in the environment requires flexibility. Researchers (Kashdan and Rottenberg, 2010) have argued that a fundamental component of health is psychological flexibility and have suggested that HRV may provide the psychophysiological foundation for such flexibility (Friedman and Thayer, 1998; Kashdan and Rottenberg, 2010). By contrast, chronic reductions in HRV are associated with psychophysiological rigidity, dysregulation of a variety of allostatic systems, and increased risk for morbidity and mortality (see Kemp and Quintana, 2013 for review). The goal of the present paper is to determine whether specific subtypes of depression display more robust alterations in heart rate and commonly reported measures of HRV relative to controls.

In the resting state, the heart is under tonic inhibitory control by the vagus, yet measures of HRV are a more specific marker of vagal function than heart rate (Saul, 1990; Reyes Del Paso et al., 2013). That said, different measures of HRV provide information on different physiological control mechanisms (Reyes Del Paso et al., 2013). For instance, high frequency oscillations (0.15–0.4 Hz) relate to respiratory influences, while LF oscillations (0.04–0.15 Hz) reflect mechanisms relating to blood pressure control such as the baroreflex (Reyes Del Paso et al., 2013; see also Krygier et al., 2013).

Reduced HRV was first reported in depressed patients more than two-decades ago (Carney et al., 1988), and more recently, has been shown to predict adverse cardiovascular events over a follow-up period of 3–15 years (Hillebrand et al., 2013), highlighting the importance of continued research in this area. However, not all studies (Licht et al., 2008) – including our own (Kemp et al., 2014) – have reported reduced HRV in depressed patients highlighting the complexity of this issue. Recent debate has focused on whether the mood and anxiety disorders, or their treatments are associated with reductions in vagal function (Licht et al., 2011; Brunoni et al., 2012). Increases in heart rate are usually associated with decreases in HRV, and antidepressant medications clearly adversely affect heart rate and HRV (Licht et al., 2010; Kemp et al., 2014), yet uncertainty remains over whether unmedicated depressed patients display alterations in these psychophysiological markers (Kemp, 2011, 2012; Kemp et al., 2011, 2014; Licht et al., 2011; Brunoni et al., 2012).

One potential explanation for the contradictory findings is that distinct subtypes (e.g., melancholia; Gold and Chrousos, 2002; Malhi et al., 2005) may display more robust alterations in heart rate and HRV, yet studies are yet to determine whether this is the case. Melancholia is characterized by an over-active stress response, a loss of responsiveness to the environment, somatic symptoms (e.g., insomnia, loss of appetite), and worse depression in the morning (Gold and Chrousos, 2002). In addition, patients with melancholia display greater cognitive impairment relative to those without such features (Quinn et al., 2012a). There are several reasons to expect that HRV will be reduced in depression generally, and that these effects will be greatest in those patients with melancholic depression. These include the presence of somatic symptoms, cognitive impairment, increased disorder severity, and reduced responsiveness to the environment; all symptoms that have been previously associated with HRV reductions.

Firstly, somatic depressive symptoms appear to be strongly associated with reduced HRV, at least in patients with stable coronary heart disease (de Jonge et al., 2007). Secondly, there is a body of evidence linking cognitive function and executive function in particular to HRV (see Thayer et al., 2009 for review; Kemp et al., unpublished findings). Participants with high HRV have been shown to perform better on executive function tasks relative to those with low HRV. These effects have been observed in a variety of populations including young men (Hansen et al., 2004) and older women (Kim et al., 2006), while performance on cognitive tasks has been shown to improve with aerobic exercise (Hansen et al., 2004; Albinet et al., 2010). Thirdly, melancholic depression is generally a more severe form of depression – although differences between melancholia and non-melancholia are more than simple variation on severity (Quinn et al., 2012b) – and reductions in HRV are correlated with increasing depression severity (Kemp et al., 2010). Fourthly, the capacity to adequately respond to change in the environment requires flexibility – melancholic patients are less responsive – and HRV may provide the psychophysiological foundation for such flexibility (Thayer and Lane, 2000; Kashdan and Rottenberg, 2010). Finally, it is notable that stimulation of the left vagus nerve is a promising, alternative treatment for treatment resistant depression (Mayberg et al., 2006), further highlighting a role for impaired vagal function in MDD.

We have reported reductions in MDD patients across time-, frequency- and non-linear domain measures of HRV (Kemp et al., 2012; see also Brunoni et al., 2013), findings associated with a small-to-moderate effect size, replicating findings we reported in an earlier meta-analysis (Kemp et al., 2010). One of our previous studies (Kemp et al., 2012) further highlighted that reductions in HRV were greatest in MDD patients with comorbid generalized anxiety disorder, findings associated with a large effect size. Confirming these findings, we recently reported, in an independent Brazilian sample, that only those with generalized anxiety disorder display robust, though small, increases in heart rate and decreases in HRV after controlling for confounding variables using propensity scores (Kemp et al., 2014), a novel approach that allows for more appropriate control of confounders, and a technique we employ in the present study. Here we explore the impact of two major subtypes of depression – melancholia and non-melancholia – relative to controls, hypothesizing that patients with melancholic depression rather than non-melancholic depression will display robust decreases in HRV, relative to healthy controls, after controlling for major confounding variables including disorder severity (Kemp et al., 2010) and comorbid anxiety (Kemp et al., 2012; Alvares et al., 2013; Chalmers et al., 2014). Here we report on a variety of HRV measures to examine the which findings are robust across different measures. On the basis of parallel lines of evidence highlighting a major role for high heart rate in future morbidity and mortality (Fox et al., 2007; Lemogne et al., 2011; Åberg et al., 2014), we also examined the impact of depression on heart rate, expecting robust increases in heart rate in melancholia.

## MATERIALS AND METHODS

### PARTICIPANTS

A total of 72 patients with a primary diagnosis of MDD and 94 age- and sex-matched controls were included in this study. Participants in this study were recruited from the community as part of case-control study conducted in 2006 and 2007 (Kemp et al., 2012). Exclusion criteria included a history of brain injury (causing loss of consciousness for 10 min or more), neurological disorder, other serious medical condition, or substance abuse or dependence for >1 year. All participants were medication free for at least five half-lives. Our study was approved by the University of Sydney, Sydney West Area Health Service, University of Adelaide and Flinders University human research ethics committees, and written informed consent was obtained from participants in accordance with National Health and Medical Research Council guidelines.

### PROCEDURES

Depressed participants were diagnosed with MDD and categorized with or without melancholic symptoms by trained and supervised research officers using the Mini International Neuropsychological Interview (MINI; Sheehan et al., 1998), a structured psychiatric interview based on the Diagnostic and Statistical Manual of Mental Disorders, Fourth Edition (DSM-IV) criteria. Control participants were excluded if they self-reported a history or presence of psychiatric illness; they were also screened using the MINI. Depression severity was assessed using the 17-item structured interview guide for the Hamilton Depression Rating Scale (SIGH-D; Hamilton, 1960; Williams, 1988) and psychomotor disturbance was measured by the CORE assessment of psychomotor change (CORE; Hickie, 1996). The self-report, Depression, Anxiety and Stress Scale (DASS; Lovibond and Lovibond, 1995) was completed by participants at the completion of the clinical interview. The DASS depression subscale is compatible with DSM-IV criteria of mood disorders, the Anxiety scale, with symptom criteria of panic disorder and PTSD, and the Stress scale, with a diagnosis of generalized anxiety disorder.

Participants were seated in a sound and light controlled room at 24°C and two 2-min electrocardiogram (ECG) recordings were collected during resting state. The ECG recording disc was positioned on the inside of the left wrist, positioned at the radial pulse, relative to a common ground and referenced to two sites: Erbs point (located two thirds distant from midline on the clavicle) and C7 (the seventh Cervical vertebra; most pronounced transverse process). Both reference sites are positioned directly above bone and serve as relatively muscle-free references. Recordings were made under these conditions as part of a standardized, psychophysiological recording protocol (Gordon et al., 2005).

Data was sampled at 500 Hz, with 22-bit resolution digitization using a Compumedics Neuroscan Nuamps amplifier and SCAN software, version 4.3. ECG was analyzed using custom-developed software to perform semi-automated pre-processing to remove noise from the ECG, allowing for the identification of the R-peaks based on established methods (Pan and Tompkins, 1985). The cleaned, N-N time-series for each participant was then imported into Kubios (version 2.0, 2008, Biosignal Analysis and Medical Imaging Group, University of Kupio, Finland, MATLAB) from which measures of heart rate and HRV were calculated based on established guidelines (Electrophysiology TFotESoCtNASoP, 1996).

### HEART RATE MEASURES

Heart rate and its variability during the resting-state are under tonic inhibitory control by the parasympathetic (vagal) nervous system (Thayer et al., 2009). It is in this regard that we refer to resting-state heart rate and HRV as surrogate measures of vagally mediated cardiac activity, although HRV measures are more pure (Saul, 1990), yet complex (Picard et al., 2009), indicators of vagal activity. HRV measures comprised time-domain estimates, including the standard deviation of N–N intervals (SDNN) and the square root of the mean squared differences between successive N–N intervals (RMSSD). SDNN is a commonly reported time-domain measure reflecting all the cyclic components responsible for variability in a recording (Electrophysiology TFotESoCtNASoP, 1996). RMSSD is a stable, time-domain index less affected by changes in breathing frequency (Penttilä et al., 2001). We also examined frequency-based estimates using the FFT method including the absolute power of high frequency (HF, 0.15–0.4 Hz) and low frequency (LF, 0.04–0.15 Hz). HF relates to respiratory influences, while LF provides information about baroreflex function (Goldstein et al., 2011). The standard deviation of the Poincaré plot perpendicular to the line of identity (PCSD1), a non-linear measure of HRV, was also calculated. We have previously reported that non-linear domain measures of HRV may be more sensitive to group differences (Kemp et al., 2010). Heart rate displays complex non-linear dynamic behavior, rather than regular, periodic oscillation (Billman, 2011), and the PCSD1 is a commonly reported, non-linear measure of short-term variability mainly caused by respiratory sinus arrhythmia (Tarvainen and Niskanen, 2008). All HRV measures were log transformed to normality.

### DATA PROCESSING AND STATISTICAL ANALYSIS

All statistical analyses were performed using IBM SPSS Statistics, Version 21 with SPSS R Essentials plug-in and R statistics version 2.14.2. To avoid bias resulting from imbalance on disorder severity and comorbid anxiety – major confounding variables when seeking to compare patients with melancholia and non-melancholia – we conducted propensity score matching (PSM) using a custom designed plugin for IBM SPSS Statistics (Thoemmes, 2012). PSM involves producing a score (on the basis of entered covariates) for each participant that relates to the probability that the subject belongs to the melancholic versus non-melancholic grouping, and then matching patients in each grouping on this propensity score. If two participants have the same propensity score, then they are equally likely to have come from the same distribution (i.e., patient grouping). Therefore, selecting patients with non-melancholia that have the same propensity scores to those with melancholia, we avoid any bias resulting from an imbalance on covariates. The PSM procedure uses logistic regression to produce the propensity score, in which patient grouping is used as the outcome variable and selected covariates, as predictors. Covariates entered into PSM analysis included age, gender, depression, anxiety and stress DASS scores, SIGH-D, and MINI anxiety disorder status (yes, no). Cases with the closest score were then matched using a simple 1:1 nearest neighbor matching routine based on a ‘greedy’ matching algorithm. Balance statistics and associated graphs were inspected to confirm adequacy of the match.

As PSM requires a complete dataset without missing data, we first ran multiple imputation (MI) analysis (Schafer, 1999) in IBM SPSS Statistics to replace missing values using the automatic method. While a common approach to dealing with missing data is deleting observations with missing values, and analyzing only those participants with a complete dataset, this listwise deletion approach is problematic for at least two reasons (Barzi and Woodward, 2004; van Ginkel and Kroonenberg, 2014). Firstly, it wastes data and reduces the power of analysis to determine an effect. Secondly, it may also produce biased estimates when loss of participants is systematic and not random. By contrast, MI yields estimates with good statistical properties. It uses all available data, makes less stringent assumptions about ‘missingness’ and pools plausible complete versions of an incomplete dataset into one analysis taking into account additional uncertainty due to missing data.

Data was missing for the following variables: depression (melancholia: 27.5%; non-melancholia: 37.5%), anxiety (melancholia: 22.5%; non-melancholia: 37.5%), and stress (melancholia: 22.5%; non-melancholia: 37.5%) from the DASS measure, MINI anxiety disorder status (yes, no; melancholia: no missing data; non-melancholia: 6.3%), and CORE total (melancholia: no missing data; non-melancholia: 3.1%) as predictors. MI procedures are appropriate for data in which up to 60% of values are missing (Barzi and Woodward, 2004). As recommended by others (Marshall et al., 2009), the imputation model used for missing data contained all variables to be subsequently analyzed including outcome variables (melancholic status in PSM, and heart rate and its variability in final analysis), variables to predict the missing data, and those variables to be imputed. Variables entered into MI analysis included: participant grouping, heart rate and HRV, depression, anxiety and stress from the DASS questionnaire, age, gender, SIGH-D, MINI anxiety disorder status and CORE total score.

Multiple imputation analysis produced 20 datasets relating to 72 MDD patients and PSM analysis was run on each of these datasets to obtain patients matched on propensity scores. The data for controls were then merged into each of the 20 datasets after which analyses were conducted on each dataset to examine group differences (MEL vs. NMEL vs. CTRL) on heart rate and HRV. As recommended previously (Hill, 2004), analysis of variance (ANOVA) analysis was carried out as a regression analysis using effect coding (Edwards, 1985; van Ginkel and Kroonenberg, 2014) so that results could then be combined according to Rubin’s rules (van Ginkel and Kroonenberg, 2014). This approach has the advantage of averaging over the results from different groupings determined using PSM on the multiply imputed datasets (Hill, 2004). Guidelines and software for carrying out these procedures and combining results for pooled estimates and statistics are available here: http://www.socialsciences.leiden.edu/educationandchildstudies/childandfamilystudies/organisation/staffcfs/van-ginkel.html. One-tailed *t*-tests are reported given specific directional hypotheses. Effect size measures (Cohen’s *d*) were also determined and Cohen’s guidelines (Cohen, 1988) for interpreting Cohen’s *d*’s (small, *d* = 0.2; medium, *d* = 0.5; large, *d* = 0.8) were followed. Cohen’s *d* statistics were calculated using an online calculator available here: http://www.uccs.edu/$\sim $lbecker/

## RESULTS

### PARTICIPANT CHARACTERISTICS

Participant characteristics are reported in **Table 1**. No age or gender differences were observed, however, all groups differed on depression, anxiety and stress scales (Tukey’s HSD *p* < 0.05), highlighting the need for PSM of patients in melancholia and non-melancholia patient groupings.

**Table 1:** Participant characteristics: mean (*M*) ± standard error (SD).^**1**^

	CTL (*M* ± SE)	NMEL (*M* ± SE)	MEL (*M* ± SE)	*p*-value^2^
Age	35.69 ± 1.16	34.98 ± 1.56	37.28 ± 2.07	0.657
Gender	52F/42M	17F/15M	25F/15M	0.675
SIGH-D	n/a	18.78 ± 0.68	21.50 ± 0.69	0.007
DASS-D	2.13 ± 0.25	21.70 ± 2.04	31.66 ± 1.39	<0.001^3^
DASS-A	1.19 ± 0.19	9.30 ± 1.79	15.23 ± 2.15	<0.001^3^
DASS-S	4.54 ± 0.47	19.00 ± 1.83	23.93 ± 1.88	<0.001^3^
CORE	n/a	3.45 ± 0.53	7.18 ± 0.86	<0.001^3^

*^1^*M* ± SE values based on original – not imputed data – data before propensity score matching of patient groupings; ^2^*p*-value refers to that for omnibus ANOVA, or for SIGH-D and CORE, an independent-samples *t*-test; ^3^*post hoc* tests revealed that all groups differ from each other, Tukey’s HSD *p* < 0.05*.

### HEART RATE AND ITS VARIABILITY

**FIGURE 1:**
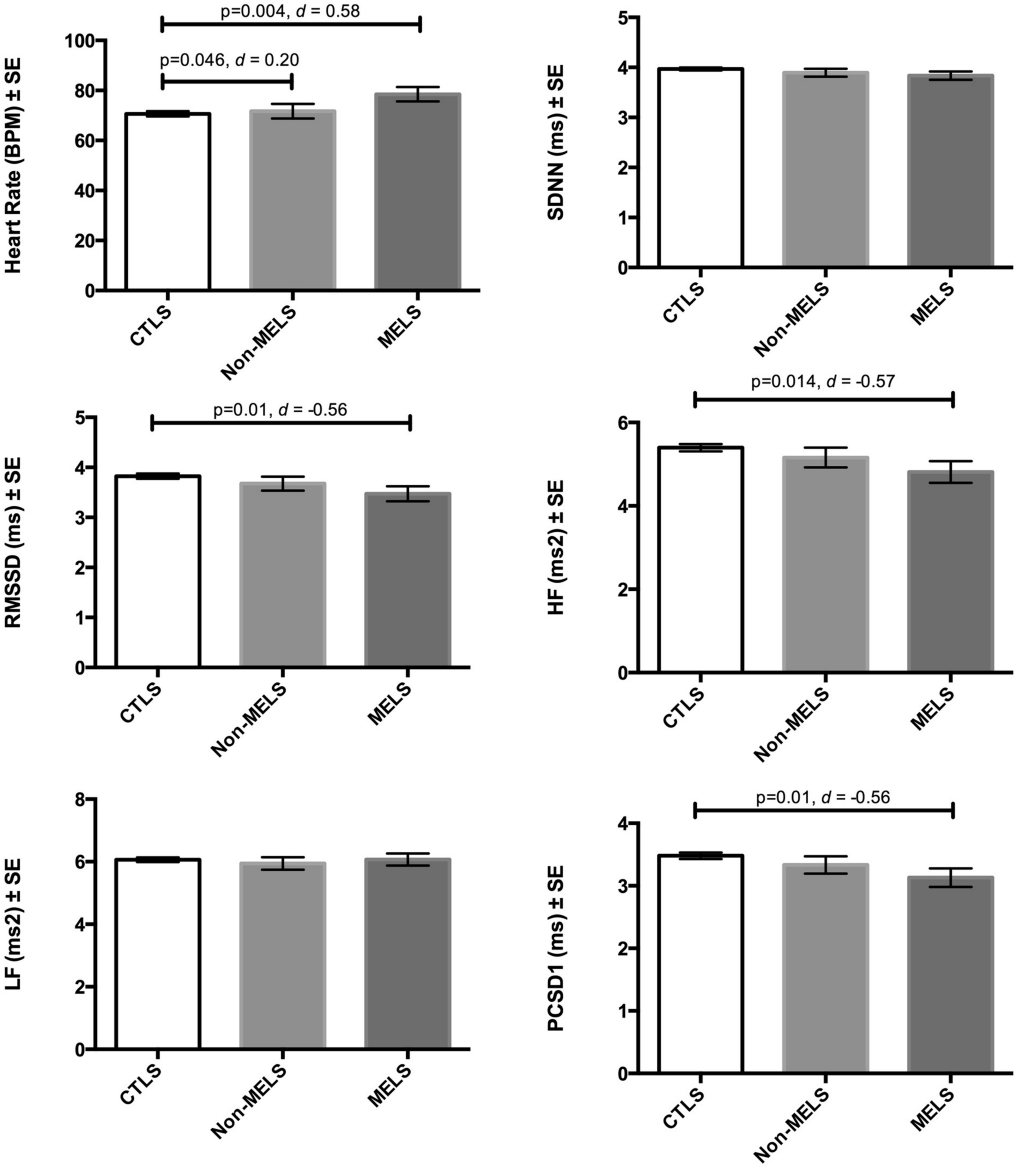
**Means and standard error for participant groupings (controls, non-melancholia, melancholia) across each of the dependent measures after controlling for age, gender, disorder severity, and comorbid anxiety disorders with propensity score matching**.

After application of PSM and pooling results according to Rubin’s rules, groups differed significantly on heart rate [*F*(2,108.25) = 3.664, *p* = 0.029], RMSSD [*F*(2,107.86) = 3.21, *p* = 0.044], HF (at trend levels) [*F*(2,102.76) = 2.966, *p* = 0.056], and PCSD1 [*F*(2,107.87) = 3.199, *p* = 0.045; **Figure 1**]. No significant differences on the combined overall test were observed for SDNN or LF. As hypothesized, heart rate was increased (by 7.85 beats per minute, BPM) in patients with melancholia – but not in those with non-melancholia – relative to controls (*p* = 0.004, one-tailed, *d* = 0.58). Also as hypothesized, HRV was decreased in patients with melancholia – but not in those with non-melancholia – relative to controls. More specifically, RMSSD (*p* = 0.01, one-tailed, *d* = 0.56), HF (*p* = 0.014, one-tailed, *d* = 0.57) and PCSD1 (*p* = 0.01, one-tailed, *d* = 0.56) were all decreased in patients with melancholia, relative to controls. No significant differences were observed between non-melancholia and controls. While patients with melancholia had higher heart rate than those with non-melancholia (by 6.75 BPM; *p* = 0.046, one-tailed, *d* = 0.20), no significant differences on measures of HRV were observed between melancholia and non-melancholia.

## DISCUSSION

The current study examined the impact of melancholia and non-melancholia on resting-state heart rate and HRV relative to controls, revealing robust alterations in patients with melancholia, but not in those with non-melancholia. These findings were associated with a moderate effect size across multiple measures (Cohens *d*’s = 0.56–0.58), providing strong evidence for an impact of melancholia on vagally mediated, cardiac function. We also observed patients with melancholia to display a higher heart rate, relative to those with non-melancholia. We and others have demonstrated that HRV is inversely associated with disorder severity (Kemp et al., 2010) and that antidepressant medications – particularly tricyclic antidepressants and the serotonin and noradrenaline reuptake inhibitors often used in more severe depressions – have adverse effects on heart rate and HRV after controlling for disorder severity (Licht et al., 2010; Kemp et al., 2014). Together, these findings suggest that alterations in melancholic patients with severe depression treated with antidepressant medications would be stronger than those effects reported here, as our patients were all unmedicated.

Here we show that resting-state heart rate is increased – by almost eight beats per minute – and HRV, decreased in patients with melancholia relative to healthy controls, findings associated with a moderate effect size. An increased resting-state heart rate may reflect vagal withdrawal without necessarily, an increase in sympathetic nervous system activity (Porges, 2011; Kemp et al., 2014). This metabolically conservative response is usually observed during environmental challenge. We have suggested (Kemp et al., 2014) that this psychophysiological state may mirror the autonomic dysregulation observed in psychiatric illness during the resting-state, and the present study highlights that this may be the case for those with melancholia in particular. As noted above however, measures of HRV are more pure indicators of vagal activity (Saul, 1990) than heart rate, which also includes sympathetic input. Differences in heart rate were also observed between those with melancholia and non-melancholia, with those with melancholia displaying a higher heart rate, a difference of 6.75 BPM, a finding associated with a small effect size (Cohens *d* = 0.20). No differences however, were observed on measures of HRV. We suggest that this finding may reflect differences in a non-vagal (perhaps sympathetic) component of cardiac function. It is possible therefore that while vagal function distinguishes between patients and controls, non-vagal components of heart rate may further distinguish between disorder subtypes.

Critically, studies have reported strong evidence for a continuous increase in risk for cardiovascular and all-cause mortality in men and women with a resting heart rate above 60 beats/min, regardless of whether individuals have a history of cardiovascular disease (Fox et al., 2007; see also Cooney et al., 2010; Saxena et al., 2013). Here we observed patients with melancholia to display a higher resting, heart rate (78.48 BPM) relative to controls (70.63 BPM) and those with non-melancholia (71.73 BPM). Other studies (Lemogne et al., 2011; Åberg et al., 2014) have reported a relationship between high heart rate and suicide. In fact, 10 additional beats per minute has been shown to increase risk of suicide by 24 to 37% over a 9 year follow-up period in adjusted models (Lemogne et al., 2011). Another study on more than 1-million, 18-year-old participants with no prior mental illness (Åberg et al., 2014) reported that poor performance on cardiovascular fitness and cognitive tests was associated with a fivefold increased risk of suicide attempt or death over a 5- to 42-year follow-up period.

In addition to increases in heart rate, we also observed decreases in all measures of HRV except for SDNN and LF in patients with melancholia relative to controls. While SDNN reflects the ‘ebb and flow’ of a variety of factors including respiration, blood pressure control mechanisms, thermoregulation and kidney functioning, RMSSD is a more specific index of vagal function that correlates highly with the high-frequency component of HRV (Kleiger et al., 2005). Recent thinking also indicates that the low-frequency component of HRV reflects blood pressure control mechanisms such as the baroreflex (Goldstein et al., 2011; Reyes Del Paso et al., 2013). It is possible therefore that SDNN from short-term recordings is less sensitive than that extracted from 24-h long recordings, and that blood pressure control mechanisms are less involved in the differences observed here between patients with melancholia and controls.

A recent meta-analysis (Hillebrand et al., 2013) reported that HRV can predict the first cardiovascular event in individuals without known cardiovascular disease over a period of 3.5 to 15 years. Cardiovascular endpoints included hospitalization for angina pectoris, myocardial infarction, congestive heart failure, arterial peripheral vascular disease, coronary revascularization, stroke and cardiovascular death. This meta-analysis (Hillebrand et al., 2013) was based on eight studies with a total of 21,988 participants without known cardiovascular disease at baseline and reported pooled relative risks for a first cardiovascular event ranging from 1.35, 1.45, and 1.32 for SDNN, low-frequency or high-frequency HRV measures respectively. The authors proposed two possible mechanisms for their findings including vagal dysregulation activating inflammatory processes culminating in cardiovascular events. The other suggested mechanism was that individuals with low HRV already suffer from subclinical or silent CVD, highlighting that reduced HRV may be both a cause and consequence of ill health. Importantly, participants in the present study were free from serious medical conditions that may have otherwise impacted on the findings reported here.

It is important to note that our findings were obtained after accounting for a variety of confounding variables including severity of depression, anxiety and stress, and number of comorbid anxiety disorders. No significant differences were observed between patients with non-melancholia and controls, suggesting that heterogeneity in patient samples may underpin some of the past null findings that have been reported in the literature. Strengths of the present study include a medication free and physically healthy sample, and application of PSM to control for a variety of confounding variables across patient groupings. It is also important to acknowledge some limitations of our study. There are potential confounding factors that we did not control for here including physical activity (Rennie et al., 2003; Soares-Miranda et al., 2014), smoking status (Sjoberg and Saint, 2011; Harte and Meston, 2014), alcohol use (Quintana et al., 2013a,b), body mass index (Britton et al., 2007; Koenig et al., 2014), and biomarkers including fasting glucose (Stein et al., 2007) and cholesterol (Britton et al., 2007; Thayer and Fischer, 2013), all of which may impact on heart rate parameters. We refer interested readers to a recent review of the various issues that researchers should consider when collecting measures of HRV (Quintana and Heathers, 2014). Regardless, we note here that many of these factors known to impact on heart rate parameters are also observed in patients with mood disorders, highlighting the importance of cardiovascular risk reduction strategies in such patients, and on the basis of the present study, those with melancholic symptoms in particular.

In conclusion, we report here that patients with melancholia – but not non-melancholia – display robust reductions in resting-state HRV relative to controls. This finding was observed even when applying PSM, a relatively novel technique to ensure that patient groupings did not differ on important confounds including age, gender, severity of depression, anxiety and stress, and comorbid anxiety disorders. Reduced vagal tone has important functional significance (e.g., impaired psychological flexibility; Thayer and Lane, 2000; Kashdan and Rottenberg, 2010), and over the longer-term may lead to significant morbidity and mortality from a host of conditions (Thayer et al., 2010; Kemp and Quintana, 2013). Our study therefore, has important implications for the health and wellbeing of patients with melancholic depression. Future studies are needed to further examine what particular behavioral features are most associated with the alterations in heart rate and HRV in patients with melancholia.

## Conflict of Interest Statement

Patrick Hopkinson was an employee at Brain Resource Company when this study was conducted. Brain Resource (http://www.brainresource.com) and Johnson & Johnson Pharmaceutical Research and Development, RED Europe funded the research on which this study is based. However, funders played no role in analysis of data, interpretation of results, writing of the paper, or decision to submit for publication.

## References

[B1] Åberg M. A. Nyberg J. Torén K. Sörberg A. Kuhn H. G. Waern M. (2014). Cardiovascular fitness in early adulthood and future suicidal behaviour in men followed for up to 42 years. *Psychol. Med.* 44 779–788 10.1017/S0033291713001207 23739044

[B2] Albinet C. T. Boucard G. Bouquet C. A. Audiffren M. (2010). Increased heart rate variability and executive performance after aerobic training in the elderly. *Eur. J. Appl. Physiol.* 109 617–624 10.1007/s00421-010-1393-y 20186426

[B3] Alvares G. A. Quintana D. S. Kemp A. H. Van Zwieten A. Balleine B. W. Hickie I. B. (2013). Reduced heart rate variability in social anxiety disorder: associations with gender and symptom severity. *PLoS ONE* 8:e70468 10.1371/journal.pone.0070468 PMC3728204 23936207

[B4] Barzi F. Woodward M. (2004). Imputations of missing values in practice: results from imputations of serum cholesterol in 28 cohort studies. *Am. J. Epidemiol.* 160 34–45 10.1093/aje/kwh175 15229115

[B5] Billman G. E. (2011). Heart rate variability – a historical perspective. *Front. Physiol.* 2:86 10.3389/fphys.2011.00086 PMC3225923 22144961

[B6] Britton A. Shipley M. Malik M. Hnatkova K. (2007). Changes in heart rate and heart rate variability over time in middle-aged men and women in the general population (from the Whitehall II Cohort Study). *Am. J. Cardiol.* 100 524–527 10.1016/j.amjcard.2007.03.056 17659940 PMC11536487

[B7] Brunoni A. R. Kemp A. H. Dantas E. M. Goulart A. C. Nunes M. A. Boggio P. S. (2013). Heart rate variability is a trait marker of major depressive disorder: evidence from the sertraline vs. electric current therapy to treat depression clinical study. *Int. J. Neuropsychopharmacol.* 16 1937–1949 10.1017/S1461145713000497 23759172

[B8] Brunoni A. R. Lotufo P. A. Benseñor I. M. (2012). Are antidepressants good for the soul but bad for the matter? Using noninvasive brain stimulation to detangle depression/antidepressants effects on heart rate variability and cardiovascular risk. *Biol. Psychiatry* 71 e27–e28; author reply e29–e30 10.1016/j.biopsych.2011.08.026 22138389

[B9] Carney R. M. Rich M. W. teVelde A. Saini J. Clark K. Freedland K. E. (1988). The relationship between heart rate, heart rate variability and depression in patients with coronary artery disease. *J. Psychosom. Res.* 32 159–164 10.1016/0022-3999(88)90050-5 3404497

[B10] Chalmers J. A. Quintana D. S. Abbott M. J.-A. Kemp A. H. (2014). Anxiety disorders are associated with reduced heart rate variability: a meta-analysis. *Front. Psychiatry* 5:80 10.3389/fpsyt.2014.00080 PMC4092363 25071612

[B11] Cohen J. (1988). Set correlation and contingency tables. *Appl. Psychol. Meas.* 12 425–434 10.1177/014662168801200410

[B12] Cooney M. T. Vartiainen E. Laakitainen T. Juolevi A. Dudina A. Graham I. M. (2010). Elevated resting heart rate is an independent risk factor for cardiovascular disease in healthy men and women. *Am. Heart. J.* 159 612–619.e3. 10.1016/j.ahj.2009.12.029 20362720

[B13] de Jonge P. Mangano D. Whooley M. A. (2007). Differential association of cognitive and somatic depressive symptoms with heart rate variability in patients with stable coronary heart disease: findings from the Heart and Soul Study. *Psychosom. Med.* 69 735–739 10.1097/PSY.0b013e31815743ca 17942844 PMC2776660

[B14] Edwards A. L. (1985). *Multiple Regression Analysis and the Analysis of Variance and Covariance.* New York, NY: Freeman.

[B15] Electrophysiology TFotESoCtNASoP. (1996). Heart rate variability: standards of measurement, physiological interpretation, and clinical use. *Circulation* 93 1043–1065. 8598068

[B16] Fox K. Borer J. S. Camm A. J. Danchin N. Ferrari R. Sendon J. L. L. (2007). Resting heart rate in cardiovascular disease. *J. Am. Coll. Cardiol.* 50 823–830 10.1016/j.jacc.2007.04.079 17719466

[B17] Friedman B. H. Thayer J. (1998). Autonomic balance revisited: panic anxiety and heart rate variability. *J. Psychosom. Res.* 44 133–151 10.1016/S0022-3999(97)00202-X 9483470

[B18] Gold P. S. Chrousos G. P. (2002). Organization of the stress system and its dysregulation in melancholic and atypical depression: high vs low CRH/NE states. *Mol. Psychiatry* 7 254–275 10.1038/sj.mp.4001032 11920153

[B19] Goldstein D. S. Bentho O. Park M. Y. Sharabi Y. (2011). Low-frequency power of heart rate variability is not a measure of cardiac sympathetic tone but may be a measure of modulation of cardiac autonomic outflows by baroreflexes. *Exp. Physiol.* 96 1255–1261 10.1113/expphysiol.2010.056259 21890520 PMC3224799

[B20] Gordon E. Cooper N. Rennie C. Hermens D. Williams L. M. (2005). Integrative neuroscience: the role of a standardized database. *Clin. EEG Neurosci.* 36 64–75 10.1177/155005940503600205 15999901

[B21] Hamilton M. (1960). A rating scale for depression. *J. Neurol. Neurosurg. Psychiatry* 23 56–62 10.1136/jnnp.23.1.56 14399272 PMC495331

[B22] Hansen A. L. Thayer J. Johnsen B. H. Sollers J. J. Stenvik K. (2004). Heart rate variability and its relation to prefrontal cognitive function: the effects of training and detraining. *Eur. J. Appl. Physiol.* 93 263–272 10.1007/s00421-004-1208-0 15338220

[B23] Harte C. B. Meston C. M. (2014). Effects of smoking cessation on heart rate variability among long-term male smokers. *Int. J. Behav. Med.* 21 302–309 10.1007/s12529-013-9295-0 23397454

[B24] Hickie I. (1996). “Validity of the CORE,” in *Melancholia: A Disorder of Movement and Mood* eds Parker G. Hazdi-Pavlovic D. (New York: Cambridge University Press), 149–159.

[B25] Hill J. (2004). *Reducing Bias in Treatment Effect Estimation in Observational Studies Suffering from Missing Data (No. ISERP Working Paper 04-01)*. New York: Institute for Social and Economic Research and Policy, Columbia University. Working Papers.

[B26] Hillebrand S. Gast K. B. de Mutsert R. Swenne C. A. Jukema J. W. Middeldorp S. (2013). Heart rate variability and first cardiovascular event in populations without known cardiovascular disease: meta-analysis and dose-response meta-regression. *Europace* 15 742–749 10.1093/europace/eus341 23370966

[B27] Kashdan T. Rottenberg J. (2010). Psychological flexibility as a fundamental aspect of health. *Clin. Psychol. Rev.* 30 865–878 10.1016/j.cpr.2010.03.001 21151705 PMC2998793

[B28] Kemp A. H. (2011). Depression, antidepressant treatment and the cardiovascular system. *Acta Neuropsychiatr.* 23 82–83 10.1111/j.1601-5215.2011.00535.x

[B29] Kemp A. H. (2012). Are antidepressants good for the soul but bad for the matter? using noninvasive brain stimulation to detangle depression/antidepressants effects on heart rate variability and cardiovascular risk. *Biol. Psychiatry* 71 e29–e30 10.1016/j.biopsych.2011.11.002 22138389

[B30] Kemp A. H. Brunoni A. R. Santos I. S. Nunes M. A. Dantas E. M. Carvalho de Figueiredo R. (2014). Effects of depression, anxiety, comorbidity, and antidepressants on resting-state heart rate and its variability: an ELSA-Brasil cohort baseline study. *Am. J. Psychiatry* 10.1176/appi.ajp.2014.13121605 [Epub ahead of print]. 25158141

[B31] Kemp A. H. Quintana D. S. (2013). The relationship between mental and physical health: insights from the study of heart rate variability. *Int. J. Psychophysiol.* 89 288–296 10.1016/j.ijpsycho.2013.06.018 23797149

[B32] Kemp A. H. Quintana D. S. Felmingham K. L. Matthews S. Jelinek H. F. (2012). Depression, comorbid anxiety disorders, and heart rate variability in physically healthy, unmedicated patients: implications for cardiovascular risk. *PLoS ONE* 7:e30777 10.1371/journal.pone.0030777 PMC3280258 22355326

[B33] Kemp A. H. Quintana D. S. Gray M. A. (2011). Is heart rate variability reduced in depression without cardiovascular disease? *Biol. Psychiatry* 69 e3–e4 10.1016/j.biopsych.2010.07.030

[B34] Kemp A. H. Quintana D. S. Gray M. A. Felmingham K. L. Brown K. Gatt J. M. (2010). Impact of depression and antidepressant treatment on heart rate variability: a review and meta-analysis. *Biol. Psychiatry* 67 1067–1074 10.1016/j.biopsych.2009.12.012 20138254

[B35] Kim D. H. Lipsitz L. A. Ferrucci L. Varadhan R. Guralnik J. M. Carlson M. C. (2006). Association between reduced heart rate variability and cognitive impairment in older disabled women in the community: Women’s Health and Aging Study I. *J. Am. Geriatr. Soc.* 54 1751–1757 10.1111/j.1532-5415.2006.00940.x 17087704 PMC2276586

[B36] Kleiger R. E. Stein P. K. Bigger J. T. (2005). Heart rate variability: measurement and clinical utility. *Ann. Noninvasive Electrocardiol.* 10 88–101 10.1111/j.1542-474X.2005.10101.x 15649244 PMC6932537

[B37] Koenig J. Jarczok M. N. Warth M. Ellis R. J. Bach C. Hillecke T. K. (2014). Body mass index is related to autonomic nervous system activity as measured by heart rate variability–a replication using short term measurements. *J. Nutr. Health Aging* 18 300–302 10.1007/s12603-014-0022-6 24626758 PMC12880227

[B38] Krygier J. R. Heathers J. A. J. Shahrestani S. Abbott M. Gross J. J. Kemp A. H. (2013). Mindfulness meditation, well-being, and heart rate variability: a preliminary investigation into the impact of intensive Vipassana meditation. *Int. J. Psychophysiol.* 89 305–313 10.1016/j.ijpsycho.2013.06.017 23797150

[B39] Lemogne C. Thomas F. Consoli S. M. Pannier B. Jégo B. Danchin N. (2011). Heart rate and completed suicide: evidence from the IPC cohort study. *Psychosom. Med.* 73 731–736 10.1097/PSY.0b013e3182365dc7 22021462

[B40] Licht C. M. M. de Geus E. J. C. van Dyck R. Penninx B. W. J. H. (2010). Longitudinal evidence for unfavorable effects of antidepressants on heart rate variability. *Biol. Psychiatry* 68 861–868 10.1016/j.biopsych.2010.06.032 20843507

[B41] Licht C. M. M. de Geus E. J. C. Zitman F. G. Hoogendijk W. J. G. van Dyck R. Penninx B. W. J. H. (2008). Association between major depressive disorder and heart rate variability in the Netherlands Study of Depression and Anxiety (NESDA). *Arch. Gen. Psychiatry* 65 1358–1367 10.1001/archpsyc.65.12.1358 19047522

[B42] Licht C. M. M. Penninx B. W. de Geus E. J. C. (2011). To include or not to include? A response to the meta-analysis of heart rate variability and depression. *Biol. Psychiatry* 69 e1; author reply e3–e4 10.1016/j.biopsych.2010.06.034 20926065

[B43] Lovibond P. F. Lovibond S. H. (1995). The structure of negative emotional states: comparison of the Depression Anxiety Stress Scales (DASS) with the Beck Depression and Anxiety Inventories. *Behav. Res. Ther.* 33 335–343 10.1016/0005-7967(94)00075-U 7726811

[B44] Malhi G. S. Parker G. B. Greenwood J. (2005). Structural and functional models of depression: from sub-types to substrates. *Acta Psychiatr. Scand.* 111 94–105 10.1111/acp.2005.111.issue-2 15667428

[B45] Marshall A. Altman D. G. Holder R. L. Royston P. (2009). Combining estimates of interest in prognostic modelling studies after multiple imputation: current practice and guidelines. *BMC Med. Res. Methodol.* 9:57 10.1186/1471-2288-9-57 PMC2727536 19638200

[B46] Mayberg H. S. Nemeroff C. Krahl S. E. Mcnamara J. Frazer A. Henry T. R. (2006). VNS therapy in treatment-resistant depression: clinical evidence and putative neurobiological mechanisms. *Neuropsychopharmacology* 31 1345–1355 10.1038/sj.npp.1301082 16641939

[B47] Pan J. Tompkins W. J. (1985). A real-time QRS detection algorithm. *IEEE Trans. Biomed. Eng.* 32 230–236 10.1109/TBME.1985.325532 3997178

[B48] Penttilä J. Helminen A. Jartti T. Kuusela T. Huikuri H. V. Tulppo M. P. (2001). Time domain, geometrical and frequency domain analysis of cardiac vagal outflow: effects of various respiratory patterns. *Clin. Physiol.* 21 365–376 10.1046/j.1365-2281.2001.00337.x 11380537

[B49] Picard G. Tan C. O. Zafonte R. Taylor J. A. (2009). Incongruous changes in heart period and heart rate variability with vagotonic atropine: implications for rehabilitation medicine. *PM R* 1 820–826 10.1016/j.pmrj.2009.07.017 19769915

[B50] Porges S. W. (2011). *The Polyvagal Theory: Neurophysiological Foundations of Emotions, Attachment, Communication, and Self-regulation* 1st Edn. New York: W. W. Norton & Company.

[B51] Quinn C. R. Harris A. Felmingham K. Boyce P. Kemp A. H. (2012a). The impact of depression heterogeneity on cognitive control in major depressive disorder. *Aust. N. Z. J. Psychiatry* 46 1079–1088 10.1177/0004867412461383 23104927

[B52] Quinn C. Harris A. Kemp A. H. (2012b). The interdependence of subtype and severity: contributions of clinical and neuropsychological features to melancholia and non-melancholia in an outpatient sample. *J. Int. Neuropsychol. Soc.* 18 361–369 10.1017/S1355617711001858 22300644

[B53] Quintana D. S. Guastella A. J. McGregor I. S. Hickie I. B. Kemp A. H. (2013a). Moderate alcohol intake is related to increased heart rate variability in young adults: implications for health and well-being. *Psychophysiology* 50 1202–1208 10.1111/psyp.12134 23941125

[B54] Quintana D. S. McGregor I. S. Guastella A. J. Malhi G. S. Kemp A. H. (2013b). A meta-analysis on the impact of alcohol dependence on short-term resting-state heart rate variability: implications for cardiovascular risk. *Alcohol. Clin. Exp. Res.* 37(Suppl. 1) E23–E29 10.1111/j.1530-0277.2012.01913.x 22834996

[B55] Quintana D. S. Heathers J. A. J. (2014). Considerations in the assessment of heart rate variability in biobehavioral research. *Front. Psychol.* 5:805 10.3389/fpsyg.2014.00805 PMC4106423 25101047

[B56] Rennie K. L. Hemingway H. Kumari M. Brunner E. Malik M. Marmot M. (2003). Effects of moderate and vigorous physical activity on heart rate variability in a British study of civil servants. *Am. J. Epidemiol.* 158 135–143 10.1093/aje/kwg120 12851226

[B57] Reyes Del Paso G. A. Langewitz W. Mulder L. J. M. Roon A. Duschek S. (2013). The utility of low frequency heart rate variability as an index of sympathetic cardiac tone: a review with emphasis on a reanalysis of previous studies. *Psychophysiology* 50 477–487 10.1111/psyp.12027 23445494

[B58] Saul J. P. (1990). Beat-to-beat variations of heart rate reflect modulation of cardiac autonomic outflow. *Physiology* 5 32–37.

[B59] Saxena A. Minton D. Lee D.-C. Sui X. Fayad R. Lavie C. J. (2013). Protective role of resting heart rate on all-cause and cardiovascular disease mortality. *Mayo Clin. Proc.* 88 1420–1426 10.1016/j.mayocp.2013.09.011 24290115 PMC3908776

[B60] Schafer J. L. (1999). Multiple imputation: a primer. *Stat. Methods Med. Res.* 8 3–15 10.1177/096228029900800102 10347857

[B61] Sheehan D. Lecrubier Y. Sheehan K. Amorim P. Janavs J. Weiller E. (1998). The Mini-International Neuropsychiatric Interview (MINI): the development and validation of a structured diagnostic psychiatric interview for DSM-IV and ICD-10. *J. Clin. Psychiatry* 59 22 10.1016/S0924-9338(99)80239-9 9881538

[B62] Sjoberg N. Saint D. A. (2011). A single 4 mg dose of nicotine decreases heart rate variability in healthy nonsmokers: implications for smoking cessation programs. *Nicotine Tob. Res.* 13 369–372 10.1093/ntr/ntr004 21350044

[B63] Soares-Miranda L. Sattelmair J. Chaves P. Duncan G. E. Siscovick D. S. Stein P. K. (2014). Physical activity and heart rate variability in older adults: the Cardiovascular Health Study. *Circulation* 129 2100–2110 10.1161/CIRCULATIONAHA.113.005361 24799513 PMC4038662

[B64] Stein P. K. Barzilay J. I. Domitrovich P. P. Chaves P. M. Gottdiener J. S. Heckbert S. R. (2007). The relationship of heart rate and heart rate variability to non-diabetic fasting glucose levels and the metabolic syndrome: the Cardiovascular Health Study. *Diabet. Med.* 24 855–863 10.1111/j.1464-5491.2007.02163.x 17403115

[B65] Tarvainen M. Niskanen J. (2008). *Kubios HRV* (No. 13779), 2nd Edn, 1–53 Kuopio: Biosignal Analysis and Medical Imaging Group (BSAMIG), Department of Physics, University of Kuopio, Kuopio.

[B66] Thayer J. Fischer J. E. (2013). Heart rate variability, overnight urinary norepinephrine, and plasma cholesterol in apparently healthy human adults. *Int. J. Cardiol.* 162 240–244 10.1016/j.ijcard.2011.05.058 21641664

[B67] Thayer J. Hansen A. L. Saus-Rose E. Johnsen B. H. (2009). Heart rate variability, prefrontal neural function, and cognitive performance: the neurovisceral integration perspective on self-regulation, adaptation, and health. *Ann. Behav. Med.* 37 141–153 10.1007/s12160-009-9101-z 19424767

[B68] Thayer J. F. Lane R. D. (2000). A model of neurovisceral integration in emotion regulation and dysregulation. *J. Affect. Disord.* 61 201–216 10.1016/S0165-0327(00)00338-4 11163422

[B69] Thayer J. Yamamoto S. S. Brosschot J. F. (2010). The relationship of autonomic imbalance, heart rate variability and cardiovascular disease risk factors. *Int. J. Cardiol.* 141 122–131 10.1016/j.ijcard.2009.09.543 19910061

[B70] Thoemmes F. (2012). Propensity score matching in SPSS. arXiv:1201.6385. Available at: http://arxiv.org/ftp/arxiv/papers/1201/1201.6385.pdf

[B71] van Ginkel J. R. Kroonenberg P. M. (2014). Analysis of variance of multiply imputed data. *Multivariate Behav. Res.* 49 78–91 10.1080/00273171.2013.855890 24860197 PMC4029775

[B72] Williams J. B. (1988). A structured interview guide for the Hamilton Depression Rating Scale. *Arch. Gen. Psychiatry* 45 742–747 10.1007/s12160-009-9101-z 3395203

